# Deciphering the mechanistic involvement of PANoptosis in FC-MDD comorbidity through the gut-brain axis

**DOI:** 10.3389/fimmu.2026.1796870

**Published:** 2026-03-25

**Authors:** Yue Ma, Shujie Wang, Wenxin Wei, Cisong Cheng

**Affiliations:** Chengdu University of Traditional Chinese Medicine, School of Basic Medicine, Chengdu, China

**Keywords:** comorbidity, FC-MDD, gut-brain axis, PANoptosis, PANoptosome

## Abstract

Functional constipation (FC) is closely associated with major depressive disorder (MDD). Patients suffering from chronic constipation often develop psychological issues such as anxiety and low mood due to persistent physical discomfort. These emotional disturbances can become prolonged, ultimately progressing into depression. Patients with major depressive disorder frequently experience gastrointestinal dysfunction, including diarrhea and constipation, with constipation symptoms being particularly persistent. The co-occurrence of these conditions is extremely common in clinical practice and severely compromises patients’ physical and mental health. PANoptosis represents a pan-inflammatory form of programmed cell death (PCD). Unlike single-pathway PCD, it simultaneously engages pyroptosis, apoptosis, and necrosis within a single cell by recruiting key molecules from multiple PCD pathways, accompanied by massive inflammatory cytokine release. This review analyzes the panoptosis mechanism in FC-MDD based on bidirectional regulation of the gut-brain axis, offering a novel perspective on elucidating its comorbidity pathology. However, current evidence primarily stems from animal models and mechanistic studies, with limited direct clinical data on FC-MDD. Future efforts should validate these findings in clinical populations, explore the specific roles of PANoptosome complex subtypes, develop targeted intervention strategies, and advance basic research toward clinical translation to identify more precise therapeutic targets for FC-MDD.

## 1 Introduction

Functional constipation (FC) exhibits a highly significant pathological association with major depressive disorder (MDD) (1). Patients with FC, chronically troubled by symptoms such as difficulty passing stool and abdominal bloating, are prone to develop a range of emotional and cognitive impairments including anxiety, insomnia, and diminished attention (2). Persistent emotional disturbances serve as precursor symptoms for depressive episodes (3). MDD disrupts the neuro-endocrine-immune network through persistent low mood and abnormal stress responses, thereby impairing gastrointestinal function and significantly increasing the risk of developing FC (4).

This manifests as weakened intestinal motility, diminished smooth muscle function, and reduced rectal sensory sensitivity (5), ultimately leading to slow-transit or outlet-obstructive constipation. Clinical studies further confirm that patients with MDD and FC often exhibit more stubborn constipation symptoms, with lower response rates to conventional laxatives or antidepressant treatments and suboptimal therapeutic outcomes (6). This bidirectional association highlights the clinical specificity and complexity of FC-MDD, underscoring the urgent need for in-depth exploration of its pathogenesis to provide theoretical support for developing targeted treatment strategies.

PANoptosis, as a novel pan-inflammatory form of programmed cell death, breaks the traditional framework of single-pathway programmed cell death (PCD) research. It integrates key features of pyroptosis, apoptosis, and necroptosis, forming a unique molecular regulatory mechanism that cannot be explained by any single PCD pathway (7). The execution of PANoptosis is highly dependent on the hierarchical assembly and activation of the PANoptosome complex. Even with the absence of some components, cell death can still occur through other molecular pathways (8). This unique form of cell death can cause tissue damage and induce inflammatory responses, extensively participating in the pathological processes of various diseases such as bacterial infections, viral infections, tumors, inflammatory diseases, and neurodegenerative diseases (9). Consequently, PANoptosis is closely associated with diseases involving intestinal barrier disruption, intestinal inflammation, brain structural alterations, and central nervous system inflammation (10, 11), playing a particularly pivotal role in mediating the pathological progression of FC-MDD (10, 12).

The regulation and feedback of the gut-brain axis (GBA) represent a key mechanism in functional cerebral-medullary depression (FC-MDD) (13, 14). Dysfunction in the gut can influence central nervous system (CNS) function through neural, endocrine, and immune pathways, and vice versa (15). Therefore, this paper focuses on the GBA regulatory mechanisms in FC-MDD, systematically deciphering the molecular regulation of PANoptosis in both the gut and central nervous system. It identifies its upstream triggers and downstream pathological effects, exploring novel targeted therapeutic approaches based on this mechanism.

## 2 Molecular regulatory mechanisms of PANoptosis

PANoptosis is mediated by the PANoptosome, whose assembly and activation form the core molecular basis for PANoptosis occurrence. Recognition proteins within the PANoptosome (e.g., ZBP1, AIM2, RIPK1, NLRP12) possess highly specific pattern recognition capabilities, enabling precise detection of pathogen-associated molecular patterns (PAMPs, such as bacterial LPS, viral ZRNA) and damage-associated molecular patterns (DAMPs, such as mitochondrial DNA, HMGB1, ATP, etc.), and bind to adapter proteins like ASC through their own domains. By integrating adapter proteins that recruit core effector molecules, they form multiprotein complexes (16, 17), subsequently inducing core effector molecules of pyroptosis, apoptosis, and necroptosis (18, 19), thereby driving the PANoptosis process. The molecular mechanisms of PANoptosis involve the coordinated regulation of multiple pathways, with extensive and dynamic crosstalk between these pathways.

### 2.1 PANoptosome assembly and regulation

As a multiprotein complex, the PANoptosome integrates multiple regulated cell death pathways, rather than merely multiple inflammasomes, and mediates PANoptosis by hierarchically assembling signals from different death pathways, including the ZBP1-PANoptosome, AIM2PANoptosome, RIPK1-PANoptosome, and NLRP12-PANoptosome (20).

#### 2.1.1 ZBP1- PANoptosome

The ZBP1-PANoptosome is driven by ZBP1, which possesses two Z-nucleic acid binding domains (Zα domains) at its N-terminus. This structure enables ZBP1 to recognize both endogenous and viral Z-RNAs (21, 22). Upon activation, ZBP1 recruits downstream signaling molecules via its protein interaction domains to form a multiprotein complex, including RIPK3, caspase-8, RIPK1, ASC, caspase-6, NLRP3, and caspase-1, ultimately assembling into the ZBP1-PANoptosome (23). The ZBP1-PANoptosome is a key driver inducing PANoptosis in intestinal endothelial cells (24). In studies of Parkinson’s disease-associated depression (PDD), the ZBP1-PANoptosome was found to cause neuronal damage, thereby inducing depression-like behaviors (11).

#### 2.1.2 AIM2- PANoptosome

AIM2 is a multi-protein complex sensor that assembles the AIM2-PANoptosome. Its N-terminal HIN domain recruits the adaptor protein ASC and effector proteins pyrin, ZBP1, caspase-1, caspase-8, RIPK1, and RIPK3 by recognizing double-stranded DNA (dsDNA), such as pathogen DNA or mtDNA released from mitochondria (25). The AIM2-PANoptosome disrupts the tight junction protein ZO-1 between intestinal epithelial cells, impairing intestinal barrier function (26). It also induces neuronal death by driving PANoptosis (27).

#### 2.1.3 RPK1- PANoptosome

In the absence of transforming growth factor beta-activated kinase 1 (TAK1) or under TNF-α stimulation, RIPK1 recruits NLRP3, ASC, RIPK3, caspase-1, and caspase-8 to form the RIPK1PANoptosome (28). Targeting components of the RIPK1-PANoptosome offers novel therapeutic strategies for inflammatory diseases, sepsis, and cancer (29).

#### 2.1.4 NLRP12- PANoptosome

NLRP12 is a sensor containing leucine-rich repeat (LRR) domains that detects bacterial LPS, fungal components, and heme. It forms the NLRP12-PANoptosome complex with NLRP3, ASC, caspase1/8, and RIPK3 (30).

### 2.2 Core effector molecules of PANoptosis

Gasdermin D (GSDMD) is a key effector protein in pyroptosis. Caspase-1 cleaves GSDMD to generate GSDMD-N, which forms pores in the cell membrane, leading to membrane rupture, release of cellular contents, and induction of inflammatory factors IL-1β and IL-18 (31). Additionally, Gasdermin E (GSDME) also participates in pyroptosis (32). GSDMD is a key molecule inducing inflammatory bowel disease, impairing intestinal barrier function and exacerbating inflammatory responses (33). It also serves as a critical inducer in multiple CNS inflammatory disorders and neurodegenerative diseases (34), such as multiple sclerosis (MS) (35) and autoimmune encephalomyelitis (EAE) (36). Caspase-3/7 (cleaved caspase-3) is a key effector protein of apoptosis, induced by caspase-8. It triggers typical apoptotic morphological changes, including chromatin condensation and induction of apoptotic body formation (37). Caspase-3 serves as a key executor in various neurodegenerative diseases, including amyotrophic lateral sclerosis, Alzheimer’s disease, Parkinson’s disease, and Huntington’s disease, participating in neuronal death processes (38). MLKL (mixed lineage kinase domain-like protein) is an executor of necroptosis. After being phosphorylated by RIPK3 to form p-MLKL, it oligomerizes, inserts into the cell membrane, disrupts its integrity, and leads to cell lysis (39, 40). MLKL is associated with neuroinflammation and cognitive decline (41).

### 2.3 Crosstalk among PANoptosis molecules

In the FC-MDD model, caspase-3 expression is significantly elevated, and its activated form, cleaved caspase-3, drives neuronal apoptosis by mediating the mitochondrial apoptosis pathway (Bax/BclxL/caspase-3) (42). Simultaneously, caspase-8 acts as a pivotal molecular hub: it promotes apoptosis through the FADD/caspase-8/caspase-3 cascade (43), while also inhibiting necroptosis by cleaving RIPK1 and activating the RIPK3/MLKL pathway. However, studies have revealed that caspase-8 can also mediate pyroptosis by activating caspase-1 and secreting IL-1β (44).

This regulatory role positions caspase-8 as a pivotal player in PANoptosis.

## 3 Upstream triggering factors of FC-MDD and PANoptosis

**Figure 1:**
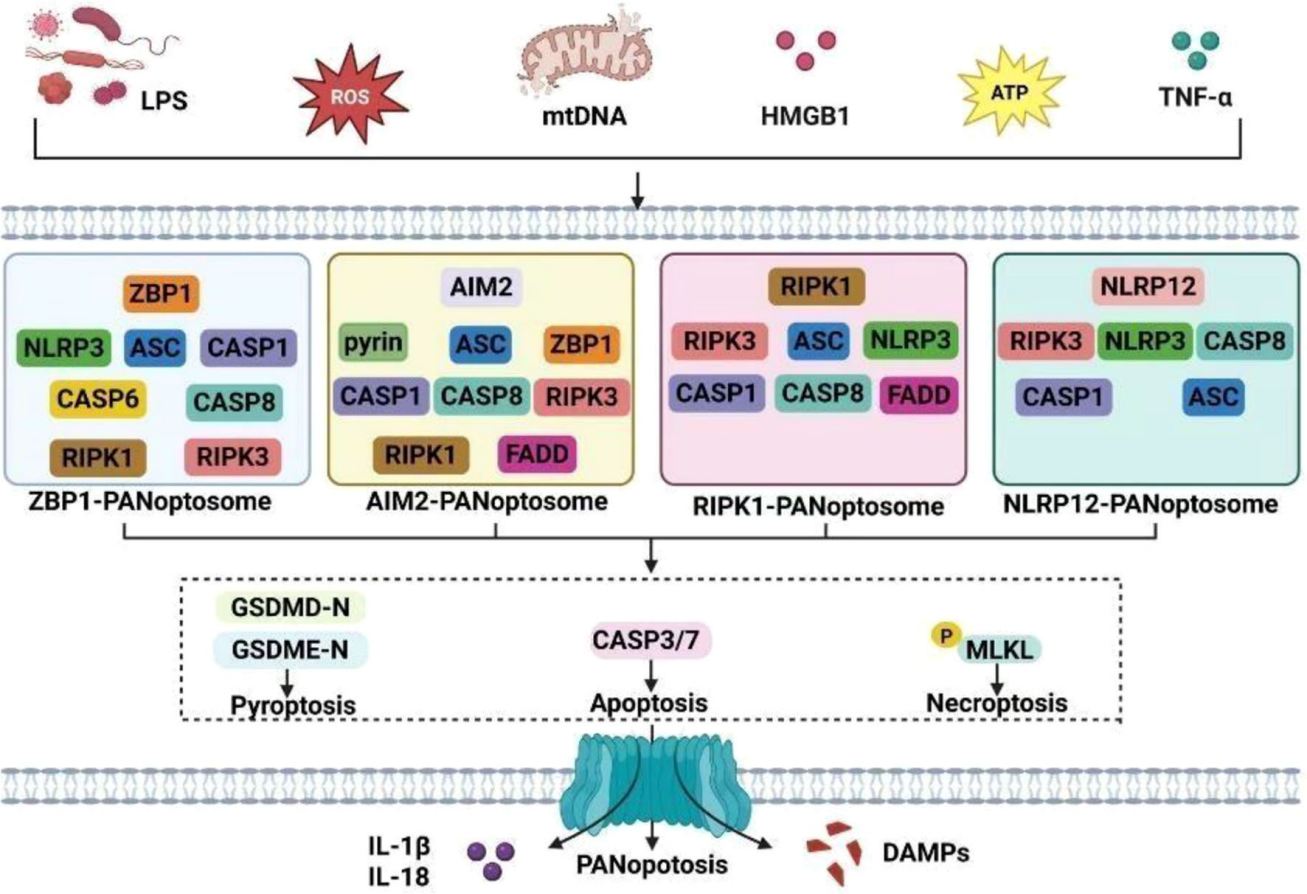
Schematic of PANoptosis induction mechanisms in the gut and brain.

The intestinal microenvironmental disruption in FC-MDD manifests as dysbiosis, compromised intestinal barrier integrity, and intestinal inflammation (45, 46). Brain structural alterations are characterized by increased blood-brain barrier (BBB) permeability, central nervous system inflammation, and neuronal and astrocytic atrophy (47, 48). The gut microenvironmental disruption in FC-MDD, alongside brain structural alterations and accompanying inflammatory responses, acts as damage-associated molecular patterns (DAMPs) and pathogen-associated molecular patterns (PAMPs), serving as key triggers for PANoptosis in both the gut and central nervous system (19, 26, 49) (**Figure 1**).

### 3.1 Disrupted gut microenvironment and PANoptosis triggering factors for brain pathological changes

The altered gut microbiota in FC-MDD is characterized by reduced relative abundance and diversity (50), decreased beneficial and anti-inflammatory bacteria, and increased harmful and proinflammatory bacteria (51, 52). Studies indicate that FC-MDD gut dysbiosis leads to increased abundance of Gram-negative pathogens (53). LPS, a core component of Gram-negative bacterial cell walls, induces mitochondrial dysfunction, leading to the release of mitochondrial DNA (mtDNA) into the cytoplasm (54). mtDNA activates the AIM2 inflammasome and forms the ZBP1PANoptosome multiprotein complex with ZBP1, thereby inducing PANoptosis (55, 56). Impaired mitochondrial function also serves as a key trigger for the intrinsic apoptotic pathway. Apoptosis released damage-associated molecular patterns (DAMPs), such as ATP and HMGB1, can further induce intestinal PANoptosis (57, 58). FC-MDD is frequently accompanied by increased ROS in both the gut and brain (59, 60). In the gut, dysbiosis and prolonged mechanical fecal compression due to constipation lead to substantial ROS production (61). On one hand, ROS can directly cause mitochondrial dysfunction, thereby inducing PANoptosis. On the other hand, ROS enhances the cleavage and activation efficiency of caspase-8, accelerating the PANoptosis process (62).

Animal studies related to depression have revealed significantly increased BBB permeability in depressive model mice (47). This increased permeability facilitates the brain penetration of gut derived LPS (63), triggering neuroinflammation and apoptosis that subsequently induce PANoptosis in the brain. Abnormal mitochondrial function in brain tissue is also closely associated with depression, encompassing mitochondrial morphological alterations, kinetic disorders, mitochondrial DNA (mtDNA) damage, and impaired respiratory chain function (64). mtDNA itself can induce PANoptosis. Within the brain, ROS induce PANoptosis by causing neuronal membrane rupture, apoptosis, neuroinflammation, and the release of ATP and HMGB1 (65). This facilitates the entry of inflammatory factors into the brain (66), triggering neuroinflammation and activating astrocytes. Consequently, the integrity of white matter structures is compromised, exacerbating depressive symptoms (67).

### 3.2 Triggers of PANoptosis in FC-MDD inflammation

Studies indicate that multiple proinflammatory cytokines are abnormally elevated in the gut, central nervous system, and peripheral blood of FC-MDD patients, particularly IL-1β, TNF-α, and IL-6 (68).

Among these, TNF-α induces the assembly of the RIPK1-PANoptosome (69), thereby activating PANoptosis. This complex integrates multiple signaling molecules including caspase-8, RIPK3, and NLRP3, synergistically initiating PANoptosis while simultaneously promoting the release of inflammatory factors such as IL-1β. This creates a positive feedback loop between inflammation and cell death.

## 4 Downstream effects of PANoptosis in FC-MDD

Constipation and depression are often accompanied by alterations in the intestinal mucosal barrier and central nervous system. PANoptosis-induced damage in both the gut and central nervous system amplifies the extent of injury, triggering more severe inflammatory responses and playing a pivotal role in the pathophysiology of FC-MDD.

### 4.1 Cross-organ transduction mechanisms of intestinal barrier damage and neuroinflammation mediated by PANoptosis

The intestinal mucosal barrier is crucial for maintaining gut health, nutrient absorption, and preventing pathogen invasion (70). Studies on FC-MDD reveal severe damage to the intestinal mucosal barrier, manifested as altered mucosal morphology, visible inflammatory cell infiltration, and reduced goblet cell numbers (71, 72). Intestinal mucosal PANoptosis induces irreversible death of intestinal epithelial cells, expanding the extent of mucosal injury. This leads to increased intestinal permeability, facilitating bacterial toxin entry into the bloodstream, activating systemic inflammation, and ultimately affecting the central nervous system (CNS) (73). Research indicates that RIPK1mediated PANoptosome activation drives epithelial cell death and barrier dysfunction. AIM2mediated PANoptosis induces intestinal epithelial cell death, disrupts intestinal barrier function, and exacerbates inflammatory responses (26).

The intestinal barrier maintains close contact with the gut microbiota on the lumenal side and interacts intimately with the enteric nervous system (ENS) on the tissue side (74). The ENS, the gut’s intrinsic nervous system composed of neurons and glial cells, regulates intestinal motility, maintains barrier integrity, and participates in immune responses (75, 76). ENS dysfunction not only reduces intestinal motility and causes barrier damage, leading to constipation, but also feeds back to the brain, forming a negative feedback loop within the gut-brain axis that plays a significant role in the pathogenesis of depression (77). Inflammatory factors released during PANoptosis, such as IL-1β, activate oxidative stress and NLRP3 inflammasome-mediated caspase-1-dependent pyroptosis and caspase-3-mediated apoptosis, leading to ENS neuronal loss and structural disruption (78).

After intestinal barrier damage, LPS and inflammatory factors entering the circulation can activate peripheral immune cells, leading to the occurrence of PANoptosis and the release of additional pro-inflammatory factors. These factors, in conjunction with LPS, act on BBB endothelial cells, inducing their PANoptosis, disrupting the tight junctions of the BBB, and increasing BBB permeability. Meanwhile, peripheral immune cells can migrate into the central nervous system through the impaired BBB, interact with microglia, and jointly activate central PANoptosis, forming a cascade transmission of “intestinal barrier damage–peripheral inflammation–BBB disruption–central inflammation.”

### 4.2 Effects of PANoptosis on the central nervous system

Within the brain, dysregulation of PANoptosis leads to neurovascular dysfunction, manifested as compromised blood-brain barrier integrity, increased vascular permeability, and abnormal cerebral blood flow regulation. These alterations may exacerbate neuronal injury and inflammatory responses, thereby impairing emotional regulation and cognitive function (79). Activation of PANoptosis disrupts the surveillance functions of microglia, including their ability to clear cellular debris and modulate neuroinflammation. Under normal conditions, microglia contribute to maintaining central nervous system (CNS) homeostasis. However, PANoptosis dysregulation shifts their function from protective to pro-inflammatory, amplifying neuroinflammation. This alteration is particularly pronounced in diseases like Alzheimer’s and Parkinson’s, accelerating neuronal damage (19). PANoptosis induces the release of the pro-inflammatory cytokine IL-1β, trapping the brain in a chronic inflammatory state (80). This inflammatory response not only directly damages neurons and glial cells but also triggers glial proliferation and neurodegeneration (81). PANoptosis leads to impaired neurotransmission and reduced neuroplasticity within the CNS (82). Neuroplasticity dysfunction represents a core mechanism in depression, contributing to dysregulation of emotional regulation (83).

In central nervous system inflammation, IL-1β released by microglia can directly induce neuronal PANoptosis (84, 85) leading to a reduction in the number of 5-HT and DA neurons (86). Furthermore, neuronal damage caused by PANoptosis releases DAMPs signals, which further amplify neuroinflammation, creating a vicious cycle of “neuroinflammation-PANoptosis-neurotransmitter imbalance.”

### 4.3 Cross-organ regulation and pathological feedback of PANoptosis in the gut-brain axis of FC-MDD

**Figure 2:**
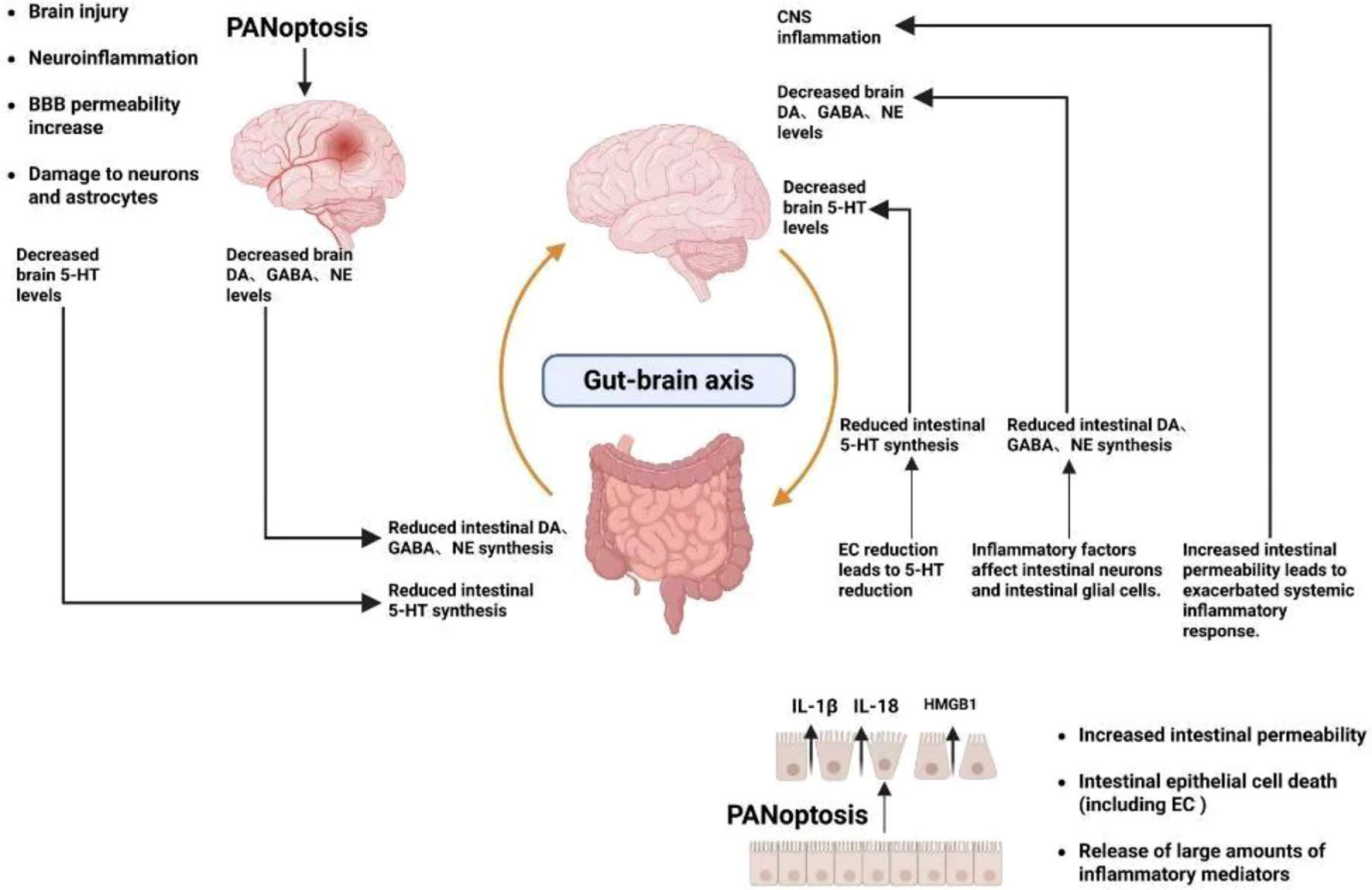
Downstream effect mechanisms of PANoptosis and regulatory mechanisms of the gut-brain axis.

The gut-brain axis (GBA) is a complex communication network that bidirectionally connects the gastrointestinal tract (GI) and the brain through neural, immune, and endocrine pathways. Dysfunction of this axis is closely associated with neuropsychiatric, neurodegenerative, and neurodevelopmental disorders (87). In the context of FC-MDD, a complex comorbidity, the key neurotransmitter hormones mediated by the GBA mainly include serotonin (5-HT), dopamine (DA), and acetylcholine (Ach), which simultaneously regulate gut motility and central mood (**Figure 2**).

Serotonin (5-HT) promotes intestinal motility and secretion while also regulating mood, alleviating anxiety, pain, and sleep disorders. It serves as a crucial intestinal hormone and neurotransmitter in functional constipation-predominant major depressive disorder (FC-MDD), modulating enteric nervous system (ENS) function and influencing the central nervous system (CNS) (87, 88).

Approximately 95% of human 5-HT is synthesized and released by enterochromaffin cells (EC), with the remaining 5% present in the brain (89). ECs reside within intestinal epithelial cells. Panoptosis in the gut leads to massive death of intestinal epithelium, including ECs, resulting in severe insufficiency of intestinal 5-HT production (90). Brain 5-HT is produced in the raphe nuclei. The proinflammatory factor IL-1β released during PANoptosis directly downregulates TPH2 mRNA and protein expression in the raphe nuclei (91). Simultaneously, it promotes 5-HT transporter (SERT) expression, accelerating synaptic 5-HT reuptake and resulting in significantly reduced brain 5-HT levels (92).

Dopamine (DA) is closely associated with gastrointestinal motility, mucosal integrity, and the gut microbiota in the intestine, while centrally it is closely linked to motor control and reward behavior (93). Dysregulation of intestinal dopamine D2 receptor signaling leads to intestinal motility disorders, which in turn induce constipation (94). The mesolimbic dopamine pathway governs reward processing. Its dysfunction diminishes responsiveness to pleasurable stimuli, resulting in anhedonia. Anhedonia constitutes one of the core symptoms of depression (95).

Acetylcholine (Ach) regulates both intestinal motility and central emotional functions. Intestinal Ach promotes smooth muscle contraction by binding to M receptors and modulates enteric nervous system (ENS) synaptic transmission by binding to N receptors (96, 97). Central ACh regulates mood and cognitive function by modulating synaptic plasticity and neurotransmitter balance (98). Impaired DA and Ach neurogenesis in the gut, coupled with ENS neuronal loss and structural disruption caused by PANoptosis, leads to insufficient intestinal DA and Ach production, consequently reducing central DA and Ach levels.

PANoptosis is not only a central hub linking intestinal barrier dysfunction, neuroinflammation, and neurotransmitter imbalance, but it also forms a bidirectional pathological feedback loop through the gut-brain axis, contributing to the further development of FC-MDD. On one hand, intestinal PANoptosis mediates gut leakage and inflammatory signals, which, through inter-organ transmission, activate central PANoptosis, inducing neuroinflammation and imbalances in 5-HT, DA, and Ach, thereby exacerbating depressive and constipation symptoms. On the other hand, brain PANoptosis-mediated central neuroinflammation aggravates the bidirectional pathological progression of FC-MDD through multiple pathways, including direct neural tissue damage, disruption of central regulatory networks, and reverse exacerbation of gut-brain axis dysregulation.

## 5 Targeted intervention strategies

Inhibitors targeting key nodes of PANoptosis show significant potential in therapeutic interventions for FC-MDD. Existing studies indicate that in mouse models of Parkinson’s disease-related depression (PDD), abnormal activation of the ZBP1-PANoptosome has been confirmed as one of the key mechanisms mediating medial prefrontal cortex (mPFC) neuron damage and inducing depression-like behaviors (11). Similarly, in pathological studies related to functional constipation (FC), activation of the ZBP1-PANoptosome pathway is also considered one of the main factors leading to intestinal epithelial cell damage, disruption of the intestinal barrier, and constipation phenotypes (10). Based on the above evidence, the ZBP1-PANoptosome may be a core molecular target connecting gut and central pathology in FC-MDD. By specifically targeting this complex, it is expected to improve both intestinal mucosal inflammatory damage and central neuronal dysfunction in patients with FC-MDD. In addition, the pan-caspase inhibitor Emricasan combined with the RIPK3 inhibitor GSK-872 can synergistically inhibit neuronal PANoptosis (99); caspase-8 can mediate both apoptosis and pyroptosis pathways, and caspase-8 inhibitors can significantly inhibit the activation of PANoptosis-related proteins (100). MLKL, as the core execution protein of necroptosis, can be effectively blocked by small-molecule inhibitors to prevent MLKL phosphorylation and membrane pore formation, reducing tissue damage (101, 102). The combined use of RIPK3 inhibitors and pan-caspase inhibitors can synergistically inhibit neuronal PANoptosis (103).

## 6 Summary and outlook

In the pathological progression of FC-MDD, dual pathological stimuli arising from gut microenvironment disruption and brain pathological alterations jointly trigger PANoptosis activation in both the gut and central nervous system through released pathogen-associated molecular patterns (PAMPs) and damage-associated molecular patterns (DAMPs) signaling. This discovery provides a novel molecular perspective and research framework for systematically elucidating the comorbidity mechanism of FC-MDD.

Based on the bidirectional regulation of the gut-brain axis in FC-MDD, PANoptosis mechanisms, PANoptosomes, and core effector molecules within the PANoptosis pathway demonstrate significant therapeutic potential. Among these, the regulatory mechanisms of the caspase family, the necroptosis effector MLKL, the pyroptosis core effector GSDMD, and GSDME warrant particular attention, as these molecules play irreplaceable roles in critical PANoptosis pathways. Future studies should validate these findings in clinical samples, delve into the specific distribution and functional differences of distinct PANoptosome complexes in the gut and central nervous system of FC-MDD, and clarify their unique roles in gut barrier damage, ENS dysfunction, neuronal injury, and the initiation of gut and central neuroinflammation. This will provide a basis for developing subtype specific targeted therapeutics. Simultaneously, focusing on key PANoptosis nodes, developing highly specific and selective small-molecule inhibitors (e.g., caspase inhibitors, MLKL phosphorylation inhibitors, GSDMD pore formation inhibitors), and integrating strategies such as gut microbiota modulation, intestinal mucosal and blood-brain barrier repair, barrier restoration, and antiinflammatory therapies to construct multi-target synergistic treatment regimens for FC-MDD.

## 7 Literature screening approach and inclusion criteria

To ensure the academic rigor, comprehensive coverage, and research transparency of this mini-review, literature screening focused on databases such as PubMed and Web of Science, retrieving original studies and high-quality reviews published from 2016 to 2025 that concentrate on FC-MDD comorbidity, depression, constipation, major depression, functional constipation, gut, central nervous system, gut-brain axis, and PANoptosis-related mechanisms. Inclusion criteria targeted literature closely related to the topic and supported by reliable evidence, prioritizing cutting-edge findings from the past five years as well as classic works in the field, while excluding literature unrelated to the topic, lacking sufficient evidence, or of low quality or redundant, ensuring the systematicity and rigor of the review.
